# Pott’s puffy tumor in coronavirus disease-2019 associated mucormycosis

**DOI:** 10.1590/0037-8682-0669-2021

**Published:** 2022-04-08

**Authors:** Ripu Daman Arora, Pugazhenthan Thangaraju

**Affiliations:** 1 All India Institute of Medical Sciences, ENT, Raipur, Chhattisgarh, India.; 2 All India Institute of Medical Sciences, Pharmacology, Raipur, Chhattisgarh, India.

**FIGURE 1:**
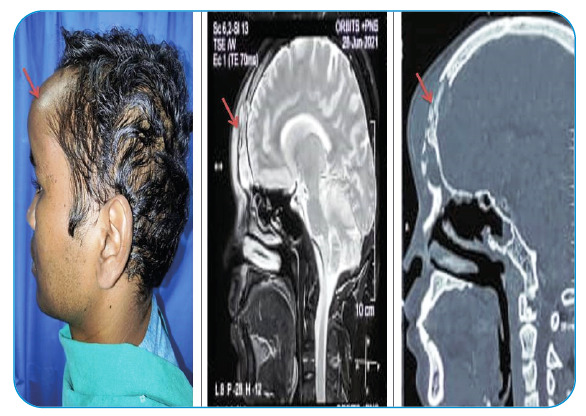
Images showing the swelling of the forehead and related computed tomography **(CT)** and contrast findings.

A 33-year-old male presented swelling of the forehead for 4 weeks. The patient was treated for moderate coronavirus disease-2019 (COVID-19) with supplemental oxygen, steroids, and remdesivir. During treatment, the patient developed periorbital swelling and was diagnosed with right sinonasal mucormycosis. The patient was fine for 1-week, after which he developed swelling in the frontal region. Boggy swelling on the forehead was 5 × 5 cm in size and nontender in nature. Vision was normal. Contrast-enhanced magnetic resonance imaging of the orbits and paranasal sinuses suggest osteomyelitis with osteonecrosis of the frontal bone associated with adjacent patchy meningitis and cerebritis, likely Pott’s puffy tumor (Figure 1). A combined endoscopic procedure with bicoronal incision frontal craniectomy with exteriorization of the frontal sinus was performed. The patient recovered without residual problems and has remained well 1-month postoperatively. Mucormycosis may develop in patients with COVID-19 due to several physiological stressors. These patients may be on high doses of steroids for an extended period in severe cases. Otherwise, they may use oxygen masks and mechanical ventilators, which facilitate fungal penetration. Furthermore, hyperglycemia serves as a source of rapid multiplication and growth^1^ ^,^ ^2^.We found no similar case in the setting of the COVID-19 pandemic, and we wanted to emphasize the development of Pott’s puffy tumor as a possible rare complication of mucormycosis. A case of aspergillosis has been reported^3^. Pott’s puffy tumor in the setting of COVID-19 and mucormycosis is unusual. Early diagnosis and prompt treatment will help patients recover from any critical consequences.
